# A real-time automated bone age assessment system based on the RUS-CHN method

**DOI:** 10.3389/fendo.2023.1073219

**Published:** 2023-03-15

**Authors:** Chen Yang, Wei Dai, Bin Qin, Xiangqian He, Wenlong Zhao

**Affiliations:** ^1^ College of Medical Informatics, Chongqing Medical University, Chongqing, China; ^2^ Medical Data Science Academy, Chongqing Medical University, Chongqing, China; ^3^ Chongqing Engineering Research Center for Clinical Big-Data and Drug Evaluation, Chongqing, China; ^4^ Department of Radiology, National Clinical Research Center for Child Health and Disorders, Ministry of Education Key Laboratory of Child Development and Disorders, Chongqing Key Laboratory of Translational Medical Research in Cognitive Development and Learning and Memory Disorders, Children’s Hospital of Chongqing Medical University, Chongqing, China

**Keywords:** RUS-CHN, bone age assessment, deep learning, prior knowledge, real-time target detection model, real-time

## Abstract

**Background:**

Bone age is the age of skeletal development and is a direct indicator of physical growth and development in children. Most bone age assessment (BAA) systems use direct regression with the entire hand bone map or first segmenting the region of interest (ROI) using the clinical *a priori* method and then deriving the bone age based on the characteristics of the ROI, which takes more time and requires more computation.

**Materials and methods:**

Key bone grades and locations were determined using three real-time target detection models and Key Bone Search (KBS) post-processing using the RUS-CHN approach, and then the age of the bones was predicted using a Lightgbm regression model. Intersection over Union (IOU) was used to evaluate the precision of the key bone locations, while the mean absolute error (MAE), the root mean square error (RMSE), and the root mean squared percentage error (RMSPE) were used to evaluate the discrepancy between predicted and true bone age. The model was finally transformed into an Open Neural Network Exchange (ONNX) model and tested for inference speed on the GPU (RTX 3060).

**Results:**

The three real-time models achieved good results with an average (IOU) of no less than 0.9 in all key bones. The most accurate outcomes for the inference results utilizing KBS were a MAE of 0.35 years, a RMSE of 0.46 years, and a RMSPE of 0.11. Using the GPU RTX3060 for inference, the critical bone level and position inference time was 26 ms. The bone age inference time was 2 ms.

**Conclusions:**

We developed an automated end-to-end BAA system that is based on real-time target detection, obtaining key bone developmental grade and location in a single pass with the aid of KBS, and using Lightgbm to obtain bone age, capable of outputting results in real-time with good accuracy and stability, and able to be used without hand-shaped segmentation. The BAA system automatically implements the entire process of the RUS-CHN method and outputs information on the location and developmental grade of the 13 key bones of the RUS-CHN method along with the bone age to assist the physician in making judgments, making full use of clinical *a priori* knowledge.

## 1 Introduction

Bone age is the age of human skeletal development, which may more precisely reflect human body maturity than age. It is a direct indication to measure children’s physical growth and development (1). In clinical medicine, the skeletal maturity status of children is a more accurate reflection of their growth and development (2). When children’s current height and bone age are known, the final height in adulthood can be predicted with high accuracy using techniques like the standard growth curve (3, 4). In clinical practice, bone age is often determined manually using bone X-ray pictures of left-hand, with methods such as the Greulich–Pyle (GP) method (5, 6), the Tanner–Whitehouse(TW) method (7), the China-05 Standards (8), and others. Although all of these approaches can identify bone age, they are all subjective assessments that rely heavily on the experience of a competent imaging specialist.

The GP approach is based on a hand atlas that includes a series of template x-ray pictures of youngsters at various stages of skeletal maturity. The patient’s x-ray pictures are then compared to samples from the template series, and the template with the closest match is chosen as the patient’s bone age. Spampinato C et al. developed BoNet, a convolutional network structure that used an end-to-end deep learning model to predict age, and took the entire hand bone map as input (9). In 2018, LARSON et al. from Stanford University’s Department of Radiology developed a deep learning model for the automated identification of bone age based on the GP method. The model employed a deep residual network structure to achieve accuracy comparable to the clinician’s. However, it was ineffective in predicting bone age in young children under two (10). Salim I et al., 2021 proposed a two-stage bone age assessment system with a mean absolute error (MAE) of 6.38 months (0.53 years), a root mean square error (RMSE) of 8.70 months (0.73 years), and a root mean squared percentage error (RMSPE) of 2.71 (11). Lee H, et al. proposed a fully automated deep learning system for bone age assessment based on GP atlas hair, obtaining a root mean square error (RMSE) of 0.93 years for females and 0.82 years for males, using a GPU time of 1.71 s for preprocessing and 10 ms for bone age prediction (12).

The TW method is mainly divided into TW2 and TW3 (13). The technique evaluates the development of particular phalangeal bones and wrist bones or the region of interest (ROI). For each ROI, skeletal maturity scores are first obtained. Then, the total maturity score is calculated by adding these scores. Finally, this score is transformed into bone age using the maturity score and bone age correlation matrix. Son, S.J et al. automated the whole process of the TW 3 method, starting from the localization of epiphyseal or metaphyseal growth regions in 13 different bones, and the MAE and RMSE of age prediction were 0.46 and 0.62 years, respectively (14). Zhou XL et al. proposed a TW3-AI model based on the TW3 method, which first obtained key bone locations, then obtained for each key bone rating growth and development scores, and finally obtained bone age based on the total score-bone age mapping relationship, achieving a mean processing time of 1.5 ± 0.2 s and a RMSE of 0.50 for the gap between bone age and the reviewing expert (15). Zhang Y et al. proposed a new automated skeletal maturity assessment with a clinically interpretable method based on the TW3 method, with mean absolute error (MAE) of 31.4 ± 0.19 points (skeletal maturity score) and 0.45 ± 0.13 years (bone age) for the carpal bone series and 29.9 ± 0.21 points and 0.43 ± 0.17 years for the radius, ulna and short (RUS) bone series, respectively (16). Peng CT et al. proposed an automatic bone age assessment system based on a convolutional neural network (CNN) framework, using the rough and fine classification of the ROI region to evaluate maturity, with final results of 0.532 and 0.56 years of MAE (mean absolute error) for females and males, respectively (17). Guo LJ et al. proposed a new dl-based bone age assessment method based on the TW method, which extracted a limited number of regions to learn representative features of these regions of interest using deep convolutional layers (18).

Both the GP method and the TW method of bone age criteria are based on samples of Caucasus children. A meta-analysis of 35 studies based on children from various ethnic groups revealed in 2019 that the GP method of bone age assessment was inaccurate and may not be preferred for Asian populations, including the Chinese pediatric population (19). The China-05 Standards was developed with a sample of Chinese adolescent children using the concepts of the TW method. However, compared to the original TW criteria, the China-05 Standards separate the bone growth process into finer-grade criteria. It increases the range of bone age assessed to 18 years for males and 17 years for females. The RUS-CHN method is one of the China-05 Standards designed to fulfill practical needs by incorporating bone maturity indicators into the TW. The RUS-CHN method first determines the ossification centers and epiphyseal ROI of 13 key bones to determine the developmental grades. Next, the developmental grades of those 13 key bones are tabulated according to the different sexes of men and women to determine the corresponding bone maturity scores. Finally, the maturity scores of all bones are added to determine the total bone maturity scores, and the bone age is determined according to those total bone maturity scores. Li NX et al., 2022 proposed a bone age assessment system incorporating prior knowledge of RUS-CHN with a MAE of 4.44 months (0.37 years) (20).

Most contemporary bone age assessment (BAA) systems are based on the GP and TW methodologies, which are inappropriate for Chinese youngsters. Most bone age assessment (BAA) systems use direct regression with the entire hand bone map or first segmenting the region of interest (ROI) using the clinical *a priori* method and then deriving the bone age based on the characteristics of the ROI, which takes more time and requires more computation. Furthermore, as technology has advanced, cell phones have become increasingly virtual devices with low arithmetic requirements, and neural networks capable of real-time detection have evolved (21, 22). Lu KJ et al. used NanoDet as a detector for identifying and locating flames in the field of vision for model selection, achieving high accuracy (23). Qu R et al. found promising results using YOLOv5 to identify and pinpoint anomalies in COVID-19 chest radiographs (24). Yu G et al. proposed that the PP-PicoDet real-time object identification model has achieved cutting-edge results (25). Ardalan Z et al. discovered in the first phase, using a transformation learning technique, that medical images utilizing a deep learning migration learning approach performed well and used fewer computational resources and time (26). Huang GH et al. discovered that using migration learning in chest X-rays can enhance prediction capabilities and reduce computing costs (27).

**Figure 1:**
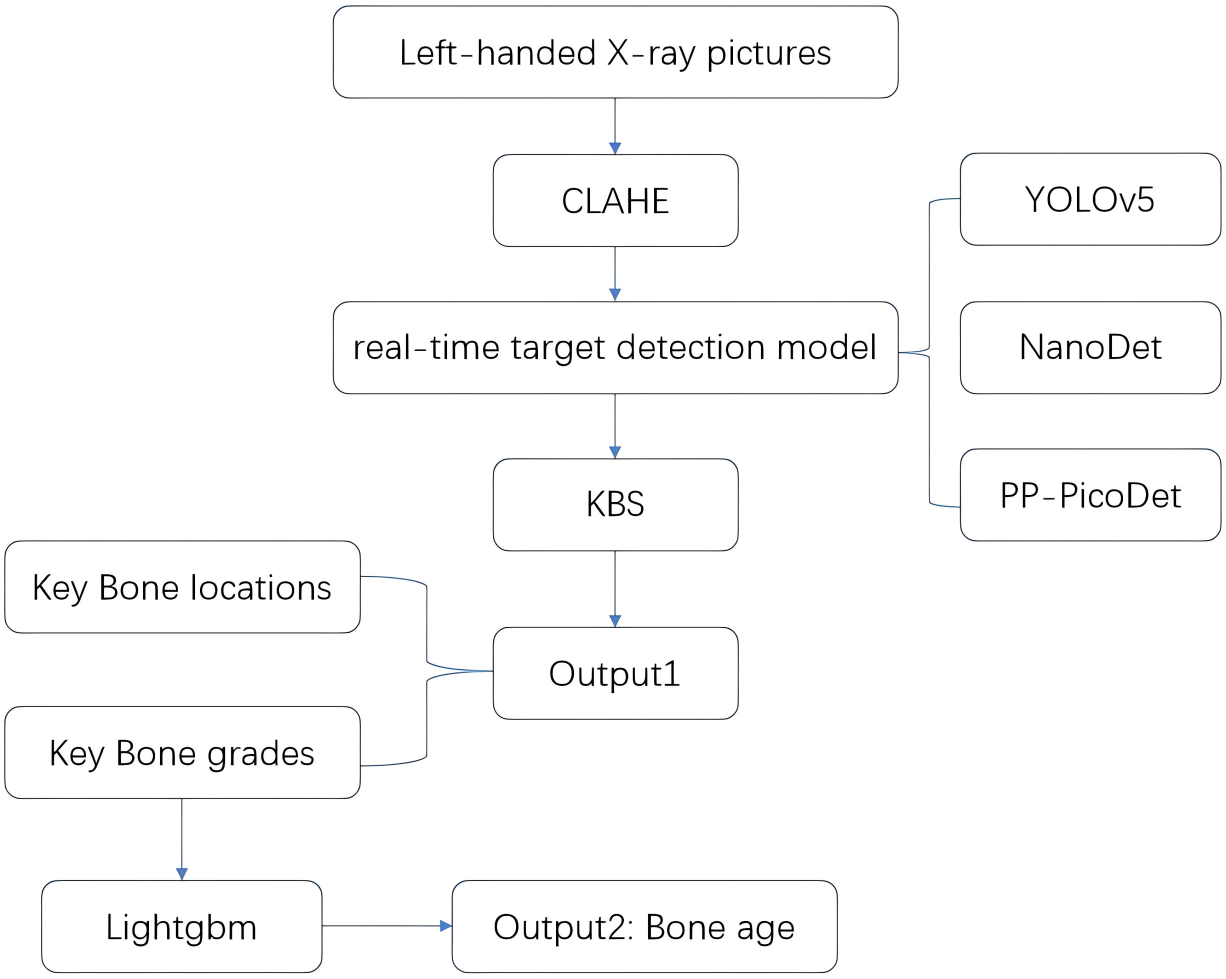
System flow chart.

In this paper, we propose a new BAA system based on the RUS-CHN method, which uses the target detection model and key bone search (KBS) to obtain the location and developmental grade of key bones in one go, and uses Lightgbm to obtain bone age. This system is a real-time bone age detection system, which can automate the whole process of the RUS-CHN method and output the location and developmental grade of 13 key bones to assist in illustrating the bone age results, which can balance the consumption of computing power resources and accuracy of detection results in a better way. The process is shown in **Figure 1**. We also created a large dataset with 4528 left-hand x-ray images and radiologists’ corresponding scores and bone age using the RUS-CHN method. Data from the test set we used were analyzed to determine the validity of the proposed BAA system.

## 2 Materials and methods

### 2.1 Patients and data

**Figure 2:**
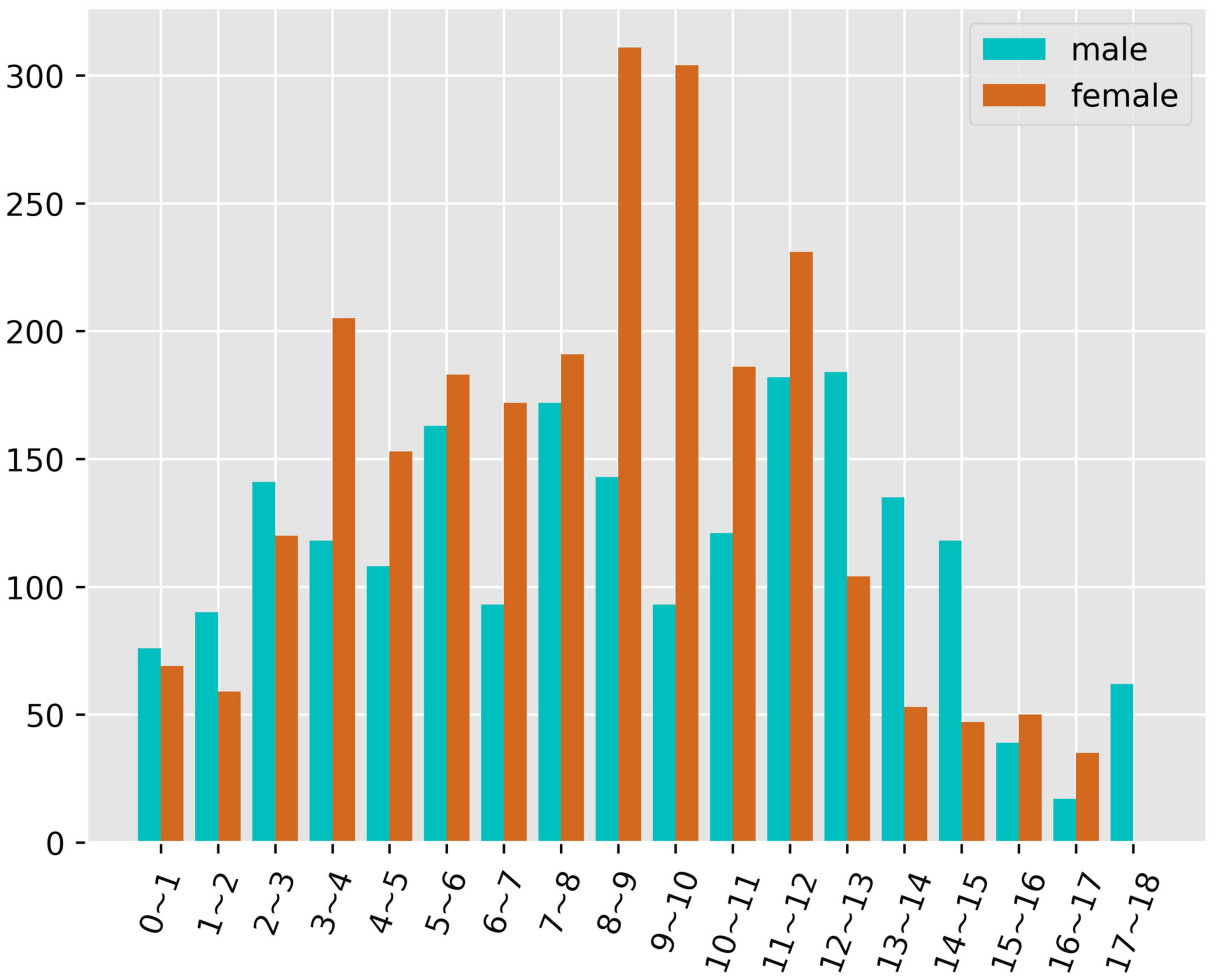
Age and gender distribution.

The study was approved by our institutional review board. Retrospective collection of 4623 posterior-anterior radiographs of the left-hand wrist of children from October 2020 to October 2021 from western China, all images were obtained from the Picture Archiving and Communication System (PACS). X-rays were numbered instead of the name, only the sex, and age were retained. Exclusion standards: 1. radiographs that were duplicates; 2. those that lack basic information, such as gender, date of birth, and shooting date; 3. radiographs that show erroneous hand placement and incomplete or variant hands; 4. Males over 18 years old and females over 17 years old. Finally, 4528 X-ray films were collected, with 2055 cases in boys and 2473 in girls. The distribution of cases in each age group for both sexes is shown in **Figure 2**, along with the number of cases in each age group.

Due to varying settings and radiation levels, the collected images were obtained using several devices with noticeable quality disparities. The DICOM format data were converted to PNG without manually segmenting the backdrop to eliminate it (12); instead, the resultant pictures were processed using Contrast Limited Adaptive Histogram Equalization (CLAHE) to ensure that they matched the original acquisition. With the help of the OpenCV (28), the CLAHE parameter, the clipLimit was 2.0, and the tileGridSize was (8, 8). The dataset was randomly partitioned into a training set (80%), a validation set (10%), and a test set (10%). Among them, the training set was 3628, the validation set was 450, and the test set was 450.

### 2.2 Skeletal maturity scores

**Figure 3:**
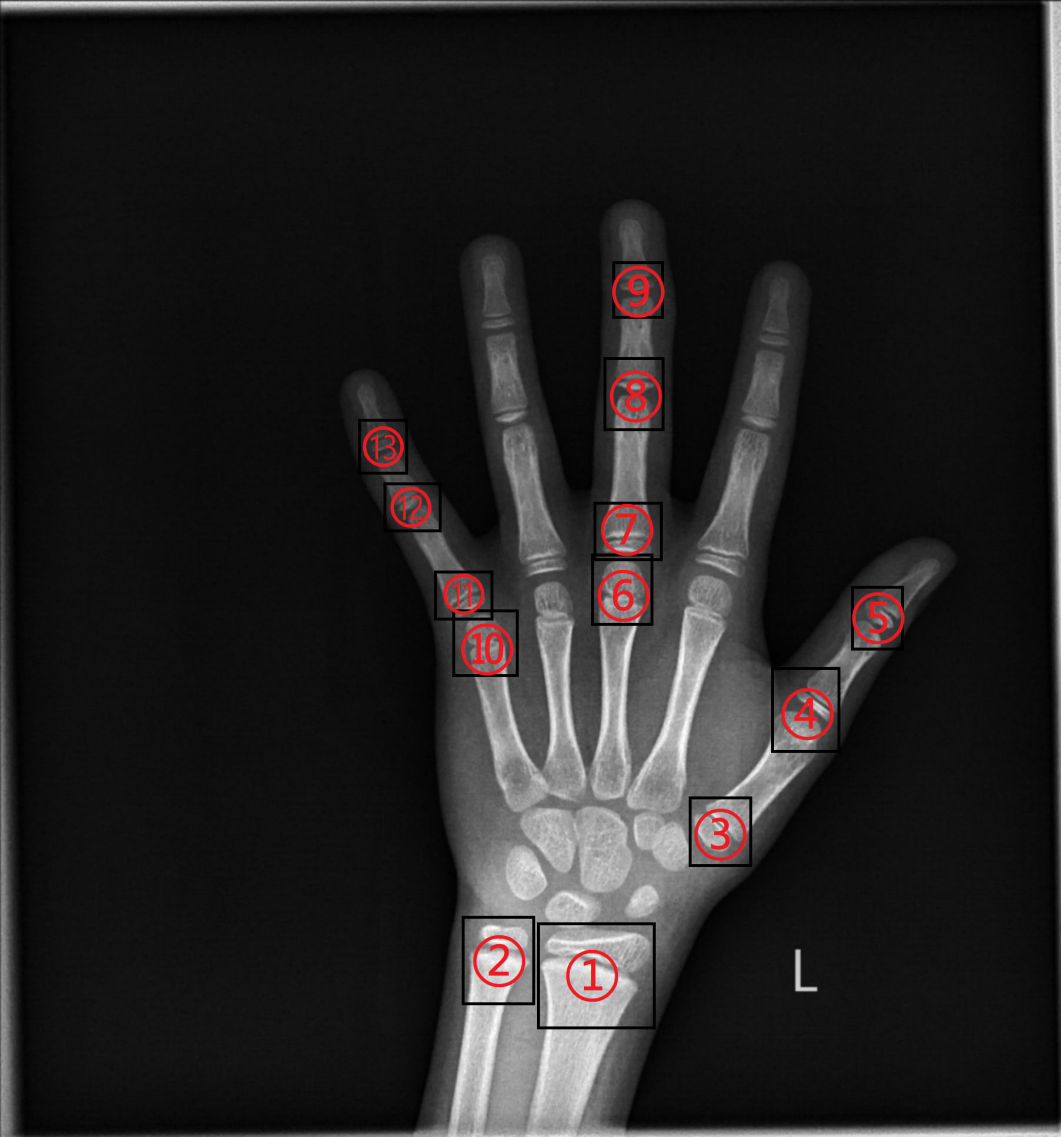
①: Radius ②: Ulna ③: First Metacarpal ④: First Proximal Phalange ⑤: First Distal Phalange ⑥: Third Metacarpal ⑦: Third Proximal Phalange ⑧: Third Middle Phalange ⑨: Third Distal Phalange ⑩: Fifth Metacarpal ⑪: Fifth Proximal Phalange ⑫: Fifth Middle Phalange ⑬: Fifth Distal Phalange.

According to the RUS-CHN method, the locations of key bones were marked. The RUS-CHN key bone location is shown in **Figure 3**. The developmental grade distribution of RUS-CHN key bones is shown in **Table 1**. Two experienced radiologists trained by the China-05 Bone Age Study Group labeled the pictures independently and graded the developmental stage of all major bones in each image using the RUS-CHN method. The key bone development grade was correct if two reviewers reported identical bone developmental stages. For ambiguous data, a third expert organized the two reviewers to reach a final result after consultation.

**Table 1:** RUS-CHN each key bone level.

		Developmental grade													
Radius	0	1	2	3	4	5	6	7	8	9	10	11	12	13	14
Ulna	0	1	2	3	4	5	6	7	8	9	10	11	12		
First Metacarpal	0	1	2	3	4	5	6	7	8	9	10	11			
Third Metacarpal	0	1	2	3	4	5	6	7	8	9	10				
Fifth Metacarpal	0	1	2	3	4	5	6	7	8	9	10				
First Proximal Phalange	0	1	2	3	4	5	6	7	8	9	10	11	12		
Third Proximal Phalange	0	1	2	3	4	5	6	7	8	9	10	11	12		
Fifth Proximal Phalange	0	1	2	3	4	5	6	7	8	9	10	11	12		
Third Middle Phalange	0	1	2	3	4	5	6	7	8	9	10	11	12		
Fifth Middle Phalange	0	1	2	3	4	5	6	7	8	9	10	11	12		
First Distal Phalange	0	1	2	3	4	5	6	7	8	9	10	11			
Third Distal Phalange	0	1	2	3	4	5	6	7	8	9	10	11			
Fifth Distal Phalange	0	1	2	3	4	5	6	7	8	9	10	11			

### 2.3 System components

The BAA system consists of two components. For the first component, we selected the real-time target detection network model (29) from YOLOv5, NanoDet, and PP-PicoDet to determine the key bone developmental grades and locations with the assistance of KBS. For PP-PicoDet, NanoDet, and YOLOv5, we selected the PP-PicoDet_s, NanoDet_plus_m_1.5x, and YOLOv5_n models, respectively. We trained these models by applying fine-tuned transfer learning using the officially provided pre-training weights. We set the hyperparameters of these models to the epoch of 300 and leave the other settings as default. In the second step, we calculate the bone age using the RUS-CHN method and Lightgbm (30) construction regression model.

### 2.4 Model acquisition

YOLOv5_n pre-training weights were available at https://github.com/ultralytics/yolov5/tree/v6.0 (accessed May 30, 2022); PP-PicoDet_s pre-training weights were available at http://github.com/PaddlePaddle/PaddleDetection/tree/release/2.5/configs/picodet (accessed May 30, 2022) NanoDet_plus_m_1.5x pre-training weights were available at https://github.com/RangiLyu/nanodet (accessed May 30, 2022)

### 2.5 Training model

**Figure 4:**
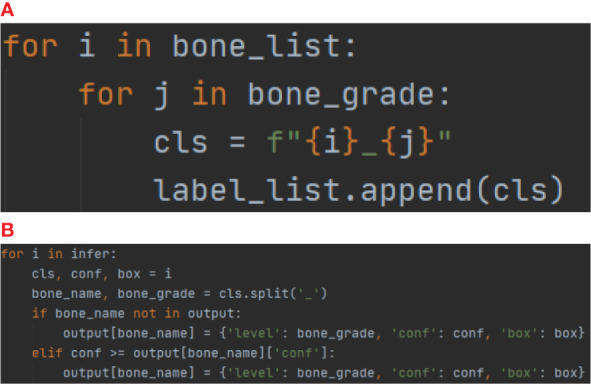
**(A)** for Encoder, **(B)** for Decoder (KBS).

**Figure 5:**
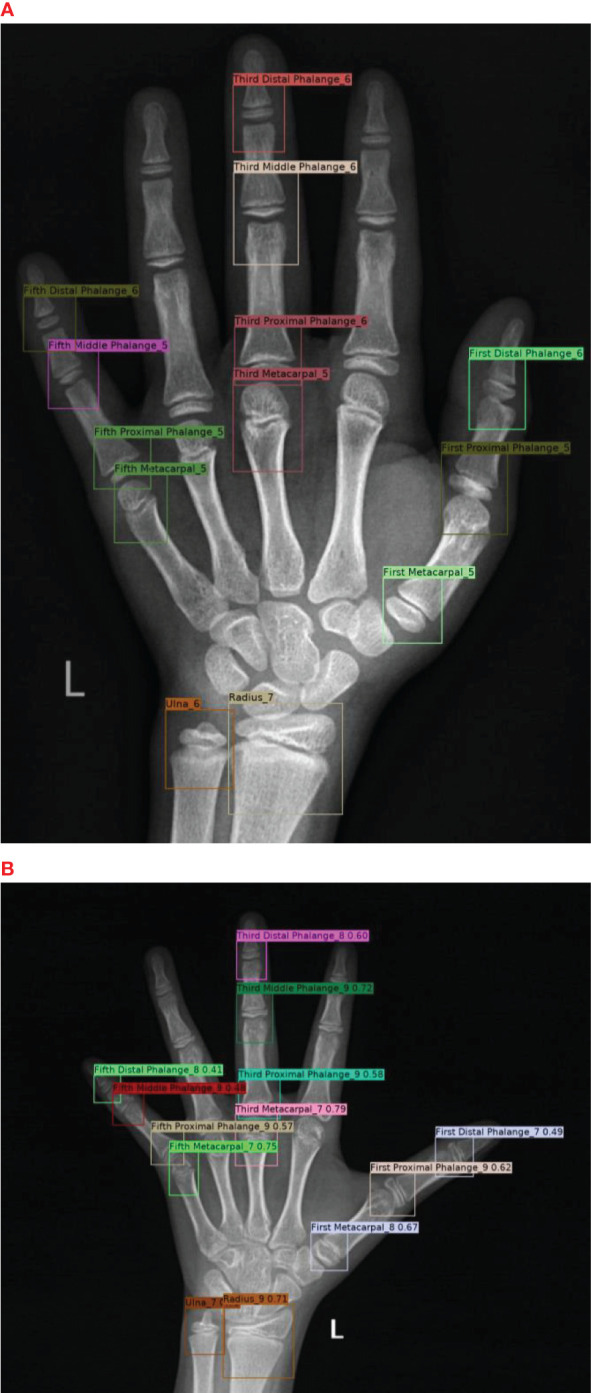
**(A)** for results after Encoding and **(B)** for model inference results after Decoding.

In the first part, Both the PP-PicoDet and NanoDet models are anchor-free models, while the YOLOv5 model employed the K-means method to obtain anchors such as [[23,24, 27,28, 26,34], [32,33, 31,41, 37,38], [38,48, 54,58, 66,69]]. The images were preprocessed before model training, including resizing the images to correspond to the size required by the model (640x640 for YOLOv5, 416x416 for PP-PicoDet, and 416x416 for NanoDet) and normalizing the images to a range of pixel values of (0, 1). And the labeling results were encoded (**Figure 4A**), where each developmental grade of each key bone was employed as a class of target (e.g., radius development grades 1 and 2 are encoded as radius_1 and radius_2, respectively), resulting in a total of 163 classes of targets—the result after coding is shown in **Figure 5A**. The mean average precision (mAP) (31) at the Intersection over Union (IOU) threshold of 0.5 was utilized as the evaluation index to assess the model effect on the validation set. The greater the mAP, the more favorable the model effect. The models that performed the best on the validation set were chosen independently to make inferences on the data from the test set. For the inference findings, we employed KBS (**Figure 4B**) to decode them rather than non-maximum suppression (NMS) (32). First, the category corresponding to the prediction result was divided into the key bone and development grade, and that grade’s confidence level and prediction box were recorded. For instance, radius_1 was divided into radius and the developmental grade 1, and that grade’s confidence level and prediction box were recorded. Likewise, radius_2 was divided into radius and the developmental grade 2, and that grade’s confidence level and prediction box were recorded. The greater confidence level was kept after comparing the two, and the kept confidence level, the developmental grade, and the prediction box were recorded for later comparison. The developmental grade, confidence level, and prediction box provided as the final outputs corresponded to the maximum confidence level for that key bone. Thirteen output results were ultimately created after performing the technique as mentioned above on all inference results to ensure that the results corresponded to a single developmental grade and prediction box for each key bone, shown in **Figure 5B**. Additionally, we applied confidence suppression to hasten to decode. Confidence suppression eliminated results that fall below the threshold for confidence without engaging in any decoding. For the selection of confidence thresholds, we chose a total of 6 different confidence thresholds of 0.0, 0.1, 0.2, 0.3, 0.4, and 0.5 for our experiments. We also contrasted the application of NMS with various confidence thresholds. The key bone developmental grades and locations were the categories we sorted the results into following the KBS. We assessed location accuracy using the Intersection over Union (IOU) (33). After plotting the confusion matrix, we calculated the key bone developmental grade classification data’s accuracy and precision (weighted average).

For the second part of the system, we used three schemes of bone age calculation:

1) We used the RUS-CHN method to calculate bone age, that was, first converting the key bone developmental grades into growth and development scores by checking the table according to male and female gender separately, then obtaining the total growth and development scores by adding all the scores, and finally obtaining the final bone age by querying the standard curve according to various male and female genders.
2) We constructed regression models using Lightgbm. The model training set data were the key bone developmental grades of the expert ratings of the training set, and the model training set labels were the bone age obtained from the key bone developmental grades of the training set based on the RUS-CHN method. The model validation set data were the key bone developmental grades of the expert ratings of the validation set, and the model validation set labels were the bone ages obtained from the key bone developmental grades of the validation set based on the RUS-CHN method. We used the optimal model of each of the three models obtained in the first part to infer the test set data to obtain three key bone inference result datasets (YOLOv5-inference-test, NanoDet-inference-test, PP-PicoDet-inference-test). The model test set data were three key bone inference result datasets (YOLOv5-inference-test, NanoDet-inference-test, PP-PicoDet-inference-test), and the model test set labels were the key bone developmental grades of the expert rating of the test set based on the bone age obtained by the RUS-CHN method. The model was trained using the training and validation sets and Optuna (34) to perform hyperparameter optimization. The best model was tested on the test set.
3) We constructed regression models using Lightgbm. We used the optimal model of each of the three models obtained in the first part to infer the training set data to obtain three key bone inference result datasets (YOLOv5-inference-training, NanoDet-inference-training, PP-PicoDet-inference-training). We used the optimal model of each of the three models obtained in the first part to infer the validation set data to obtain three key bone inference result datasets (YOLOv5-inference-validation, NanoDet-inference-validation, PP-PicoDet-inference-validation). We used the optimal model of each of the three models obtained in the first part to infer the test set data to obtain three key bone inference result datasets (YOLOv5-inference-test, NanoDet-inference-test, PP-PicoDet-inference-test). The model training set data were three key bone inference result datasets (YOLOv5-inference-training, NanoDet-inference-training, PP-PicoDet-inference-training), and the model training set labels were the key bone developmental grades of the expert rating of the training set based on the bone age obtained by the RUS-CHN method. The model validation set data were three key bone inference result datasets (YOLOv5-inference-validation, NanoDet-inference-validation, PP-PicoDet-inference-validation), and the model validation set labels were the key bone developmental grades of the expert rating of the validation set based on the bone age obtained by the RUS-CHN method. The model test set data were three key bone inference result datasets (YOLOv5-inference-test, NanoDet-inference-test, PP-PicoDet-inference-test), and the model test set labels were the key bone developmental grades of the expert rating of the test set based on the bone age obtained by the RUS-CHN method. The models were trained using the training and validation sets, hyperparameter optimization was performed using Optuna, and the best model was tested on the test set.

To this end, we used mean absolute error (MAE), root mean square error (RMSE), and root mean squared percentage error (RMSPE) as evaluation metrics (11, 35), which are defined as follows:


$$MAE=\frac{1}{n}\sum_{i=1}^{n}\left|y_{i}-{\hat{y}}_{i}\right|$$

$$RMSE=\sqrt{\frac{1}{n}\sum_{i=1}^{n}{\left(y_{i}-{\hat{y}}_{i}\right)}^{2}}$$

$$RMSPE=\sqrt{\frac{1}{n}\sum_{i=1}^{n}{\left(\frac{y_{i}-{\hat{y}}_{i}}{y_{i}}\right)}^{2}}$$

where ***n*** is the number of samples in the test set, ***y*** *_i_* is the true value, and ${\hat{y}}_{i}$ is the predicted value of the model. The smaller the value of the evaluation metric, the better the performance of the model.

For the inference time test, we systematically exported all models of the first part to Open Neural Network Exchange (ONNX) format in CPU AMD Ryzen 5600x, tested the inference time of post-processing NMS and KBS respectively, and also tested the inference time of the optimal scheme of the second part. Finally, the total elapsed time of the first part of the optimal model is tested in the GPU RTX3060 environment, including pre-processing (normalizing, resizing), the model inference, and post-processing KBS.

## 3 Results

**Figure 6:**
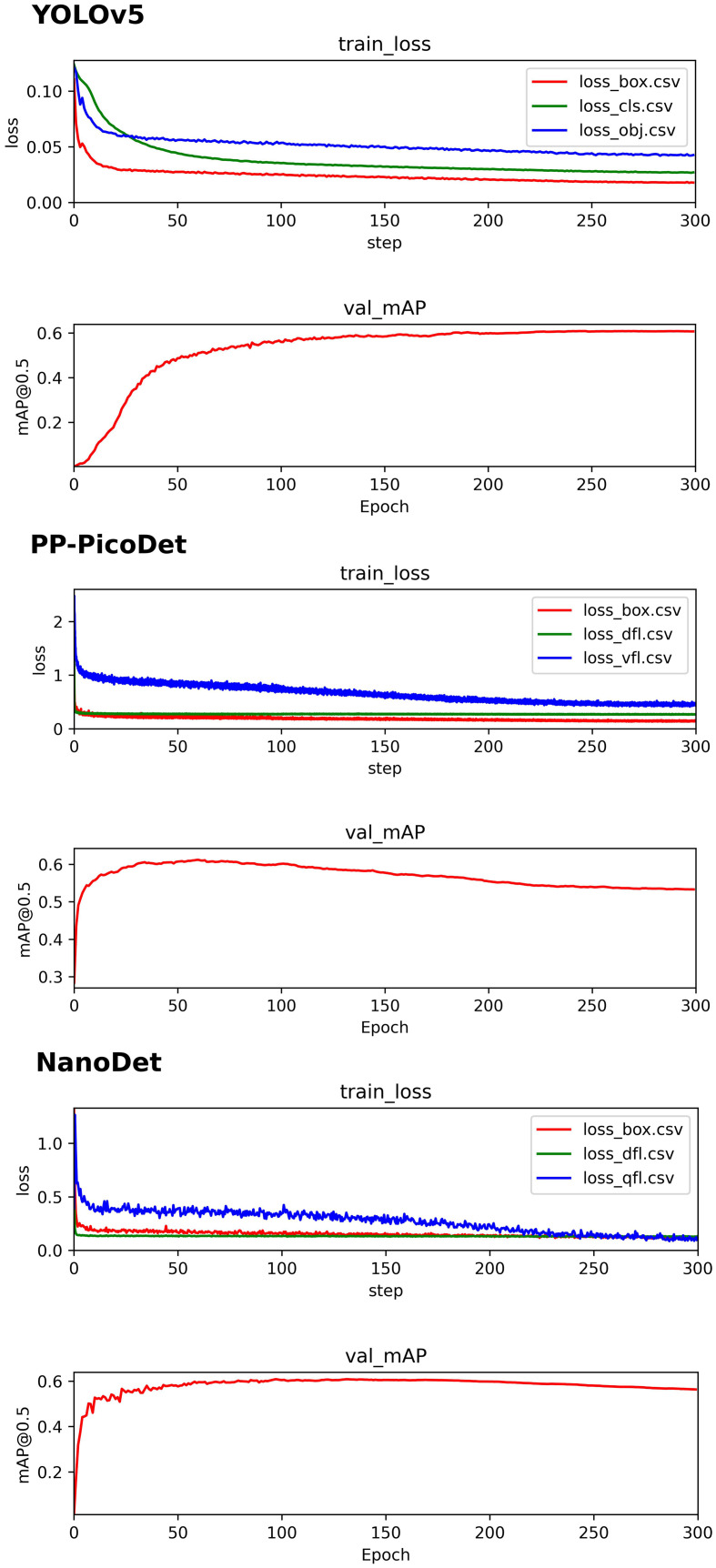
YOLOv5 、PP-PicoDet、NanoDet training process.

The initial component of the BAA system, the validation set’s best map for all three real-time models was 0.6, and the precise training procedure was depicted in **Figure 6**. On the data from the test set, we ran a KBS with various levels of confidence thresholds, and the outcomes are displayed in **Table 2**. The NanoDet inference results using the KBS did not reveal any missing key bones at confidence thresholds of 0.0, 0.1, 0.2, and 0.3. However, The NanoDet inference results using the KBS at confidence thresholds of 0.4 and 0.5, 13, and 367 key bones, respectively, were missing. The PP-PicoDet inference results using the KBS did not reveal any missing key bones at confidence thresholds of 0.0, 0.1, and 0.2. However, The PP-PicoDet inference results using the KBS at confidence thresholds of 0.3, 0.4, and 0.5, 1,2, and 56 key bones, respectively, were missing. The YOLOv5 inference results using the KBS did not reveal any missing key bones at confidence thresholds of 0.0, 0.1, and 0.2. However, The YOLOv5 inference results using the KBS at confidence thresholds of 0.3, 0.4, and 0.5, 15,106, and 296 key bones, respectively, were missing.

**Table 2:** Inference time and number of errors at different confidence levels.

Confidence threshold	YOLOv5		PP-PicoDet		NanoDet	
	Inference time (ms)	lack	Inference time (ms)	lack	Inference time (ms)	lack
**0.0**	80	0	63	0	69	0
**0.1**	33	0	57	0	61	0
**0.2**	33	0	57	0	61	0
**0.3**	33	15	57	1	60	0
**0.4**	33	106	57	2	60	13
**0.5**	32	296	57	56	59	367

**Figure 7:**
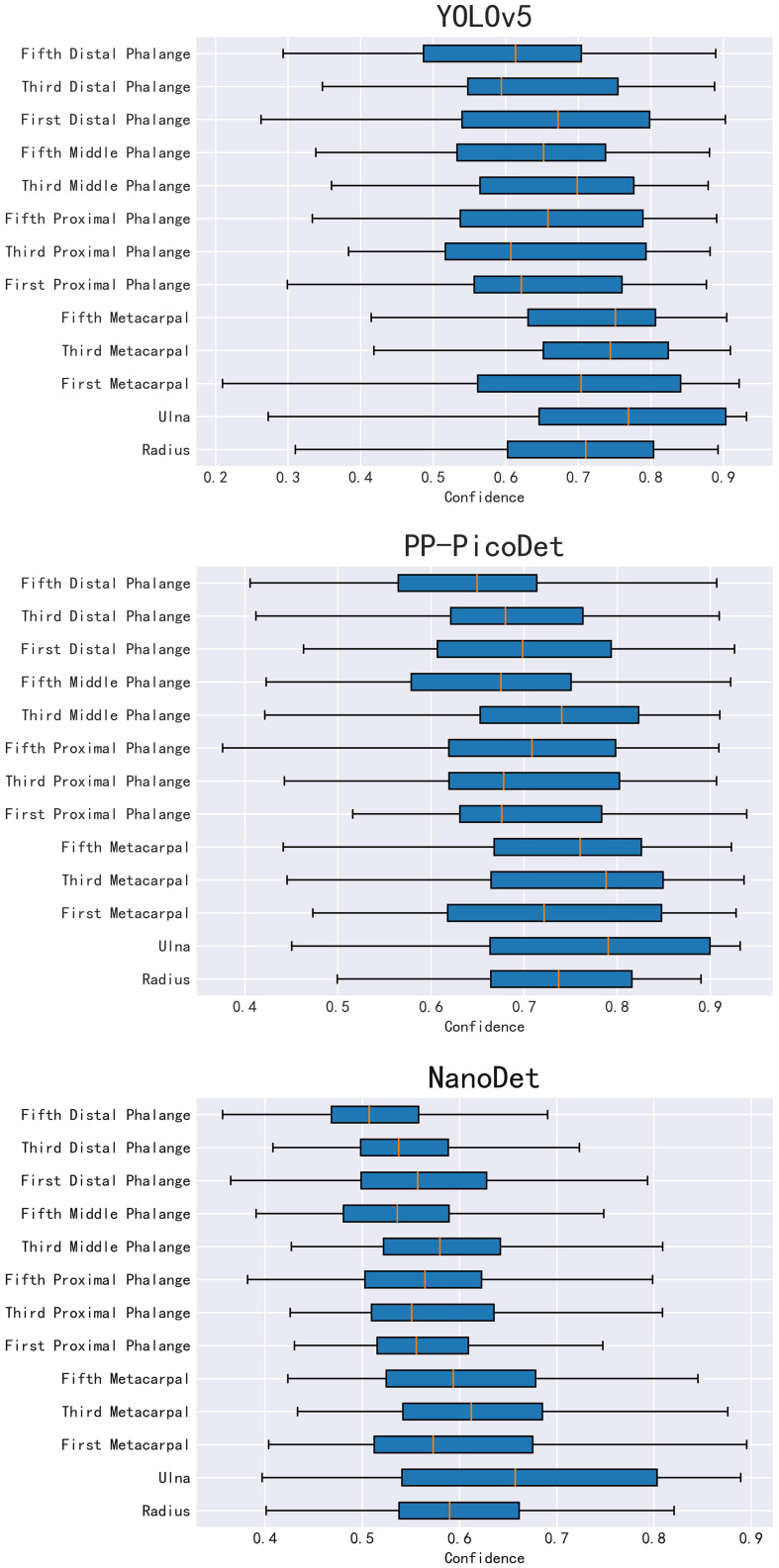
YOLOv5、PP-PicoDet 、NanoDet key bones Confidence distribution.

**Figure 8:**
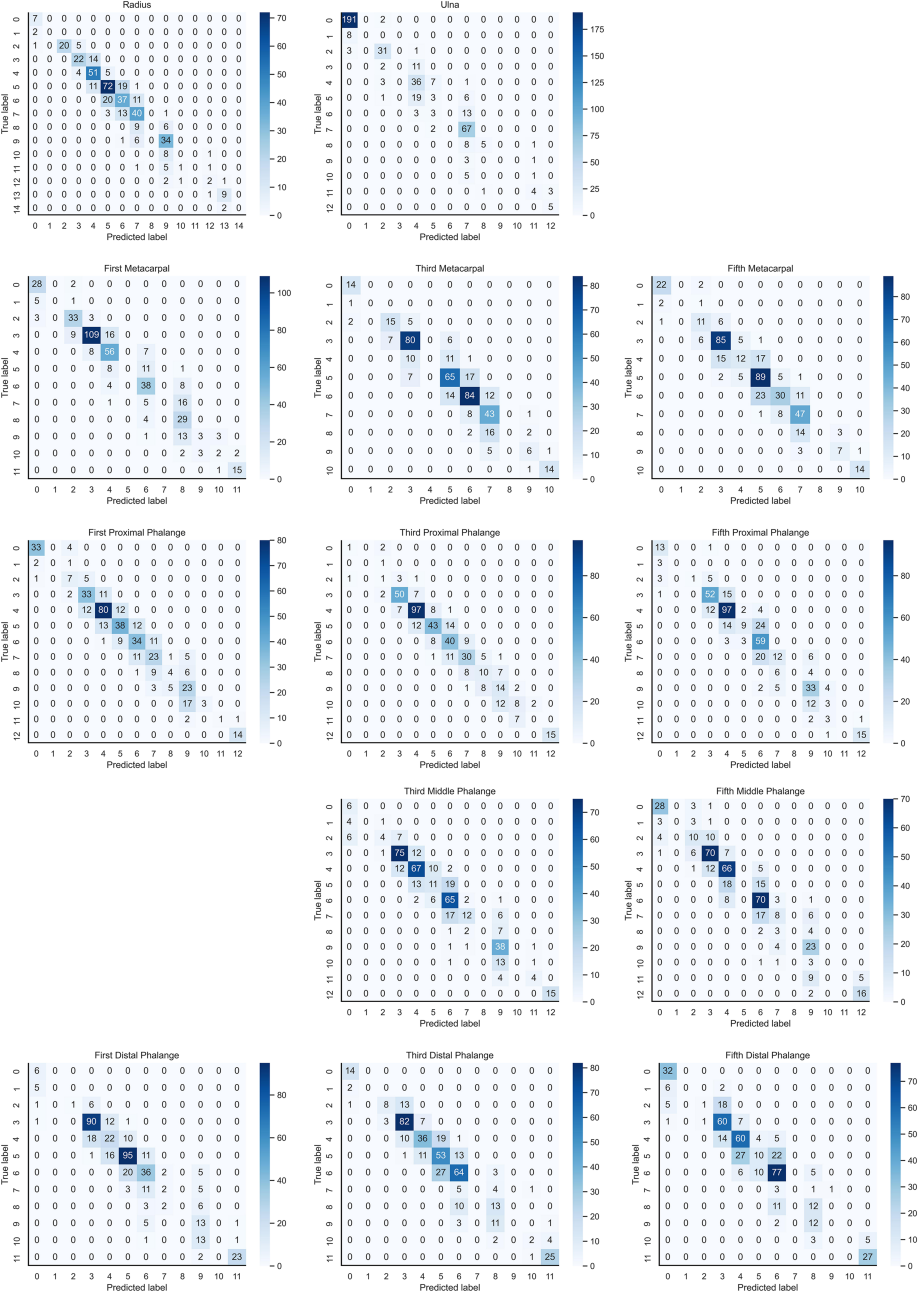
Confusion matrix for YOLOv5 inference results after KBS.

**Figure 9:**
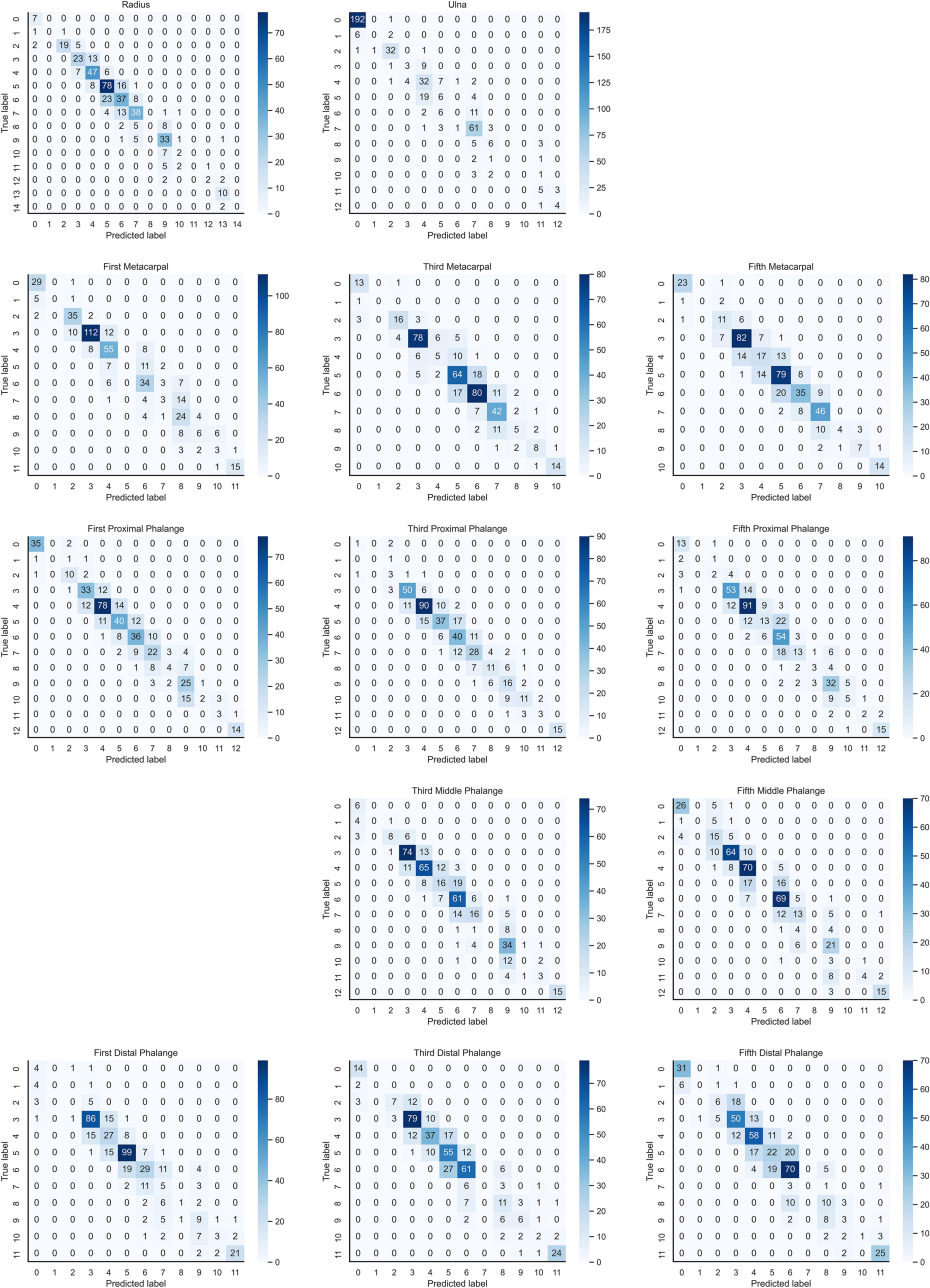
Confusion matrix for PP-PicoDet inference results after KBS.

**Figure 10:**
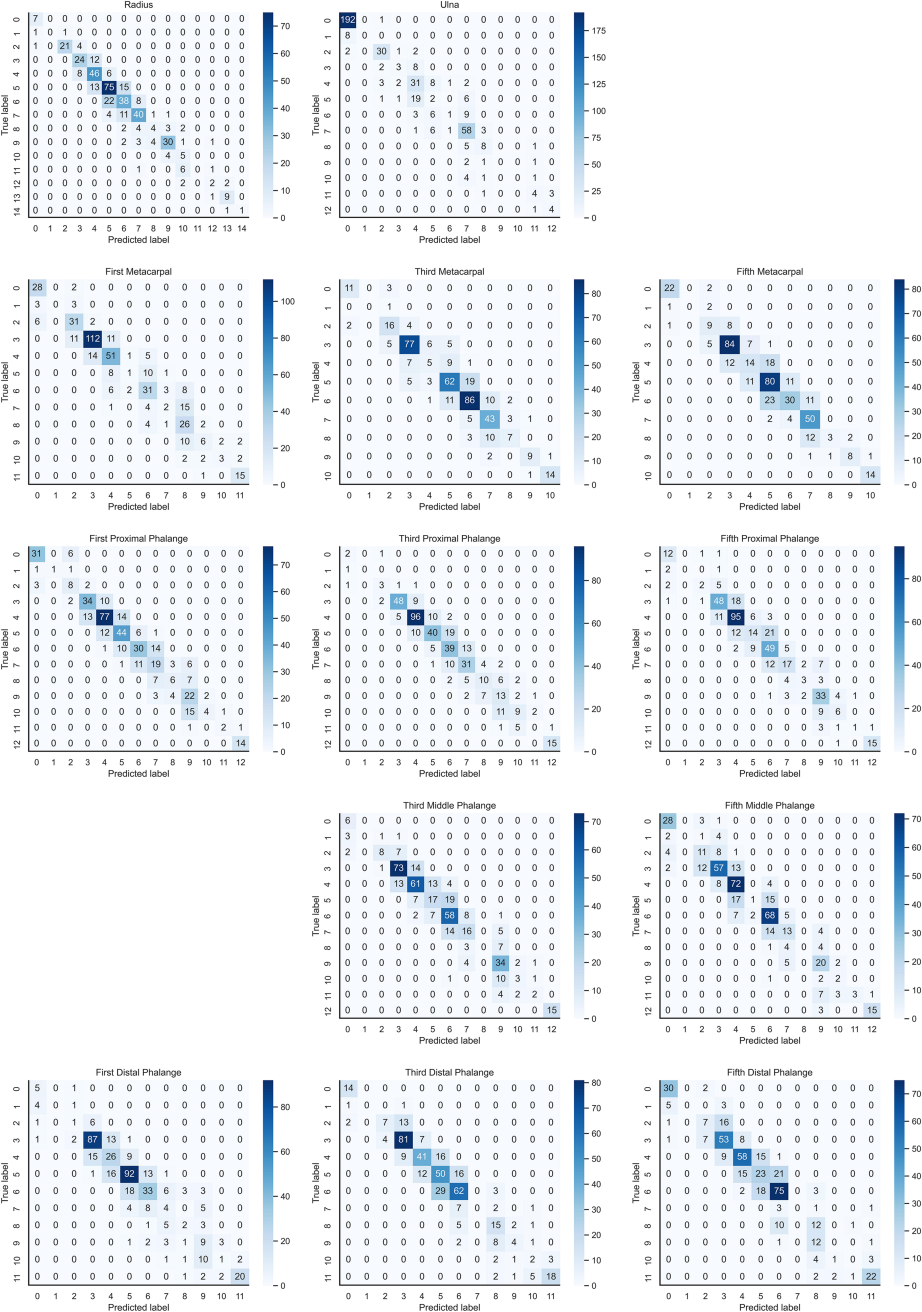
Confusion matrix for NanoDet inference results after KBS.

In the instance of employing NMS, **Table 3** shows that the key bone results were duplicated, i.e., many developmental grades and target boxes appear for one key bone when the confidence threshold was selected less. We discovered duplicate key bone results and missing key bone results as the confidence threshold rose. NMS requires much time when the confidence threshold was set to 0.0. NMS also took longer to complete than KBS for the remaining confidence scenarios. We also counted the distribution of the confidence of the results after KBS when the confidence threshold was 0.0, as shown in **Figure 7**. The three models produced good results for the outputs location evaluation findings shown in **Table 4**, with an average IOU no less than 0.9 in all key bones, indicating that the predicted and labeled positions were very similar to one another. We displayed confusion matrices for the key bone developmental grade results. See **Figure 8** for YOLOv5, **Figure 9** for PP-PicoDet, and **Figure 10** for NanoDet. Strong diagonal patterns can be seen in the three confusion matrices, implying that the labels predicted by the three models that post-processed labels with KBS were most often the correct skeletal maturity. As shown in **Table 5**, we also determined each key bone’s precision (weighted average) and accuracy for the three models.

**Table 3:** NMS experiment.

Confidence threshold	YOLOv5			PP-PicoDet			NanoDet		
	Inference time (ms)	lack	repeat	Inference time (ms)	lack	repeat	Inference time (ms)	lack	repeat
0.0	3690	0	450	293	0	450	789	0	450
0.1	47	0	450	65	1	450	93	0	450
0.2	45	0	450	65	1	359	80	0	450
0.3	44	15	450	65	1	188	78	0	450
0.4	43	106	450	66	2	104	76	13	450
0.5	42	296	450	65	56	57	74	367	450

**Table 4:** Mean Intersection over Union (IOU) results.

	YOLOv5	PP-PicoDet	NanoDet
Radius	0.93	0.93	0.93
Ulna	0.92	0.92	0.92
First Metacarpal	0.92	0.92	0.92
Third Metacarpal	0.92	0.93	0.93
Fifth Metacarpal	0.92	0.92	0.92
First Proximal Phalange	0.93	0.93	0.93
Third Proximal Phalange	0.90	0.92	0.91
Fifth Proximal Phalange	0.91	0.92	0.92
Third Middle Phalange	0.93	0.94	0.94
Fifth Middle Phalange	0.92	0.92	0.93
First Distal Phalange	0.92	0.93	0.93
Third Distal Phalange	0.92	0.93	0.93
Fifth Distal Phalange	0.90	0.91	0.91

**Table 5:** Precision (weighted average) and Accuracy of key bones.

	YOLOv5		PP-PicoDet		NanoDet	
	accuracy	Precision (weighted average)	accuracy	Precision (weighted average)	accuracy	Precision (weighted average)
Radius	0.65	0.67	0.66	0.68	0.67	0.68
Ulna	0.76	0.79	0.76	0.72	0.74	0.73
First Metacarpal	0.70	0.75	0.70	0.73	0.68	0.67
Third Metacarpal	0.71	0.75	0.72	0.71	0.73	0.73
Fifth Metacarpal	0.70	0.71	0.71	0.71	0.70	0.69
First Proximal Phalange	0.65	0.68	0.67	0.68	0.65	0.66
Third Proximal Phalange	0.69	0.68	0.68	0.69	0.68	0.68
Fifth Proximal Phalange	0.65	0.68	0.66	0.65	0.66	0.65
Third Middle Phalange	0.66	0.69	0.66	0.66	0.65	0.66
Fifth Middle Phalange	0.65	0.70	0.66	0.72	0.64	0.65
First Distal Phalange	0.64	0.68	0.63	0.64	0.62	0.62
Third Distal Phalange	0.66	0.69	0.66	0.67	0.65	0.67
Fifth Distal Phalange	0.62	0.60	0.61	0.60	0.62	0.61

The bone age calculation approach employing Lightgbm-based modeling performed better than RUS-CHN in the second portion of the BAA system. The exact outcomes of the bone age prediction utilizing the Lightgbm model are provided in **Table 6**, and scheme 3 performed better than scheme 2 when using the model. When employing KBS, the key bone developmental grades of the YOLOv5 inference results had superior accuracy than the key bone developmental grades of the PP-PicoDet and NanoDet inference results. The most accurate outcomes for the YOLOv5 inference results utilizing KBS were a MAE of 0.35 years, a RMSE of 0.46 years, and a RMSPE of 0.11, with the optimal training hyperparameters of ‘num_leaves = 2360, max_depth = 3, learning_rate = 0.18514590909895523, stopping_rounds = 300’, and the rest of the hyperparameters were default values. The most accurate outcomes for the NanoDet inference results utilizing KBS were a MAE of 0.38 years, a RMSE of 0.49 years, and a RMSPE of 0.12, with the optimal training hyperparameters of ‘num_leaves = 1960, max_depth = 3, learning_rate = 0.18804401074897045, stopping_rounds = 300’, and the rest of the hyperparameters were default values. The most accurate outcomes for the PP-PicoDet inference results utilizing KBS were a MAE of 0.39 years, a RMSE of 0.50 years, and a RMSPE of 0.12, with the optimal training hyperparameters of ‘num_leaves = 1960, max_depth=3, learning_rate = 0.18804401074897045, stopping_rounds = 300 ‘, and the rest of the hyperparameters were default values.

**Table 6:** Bone age Calculation results.

		YOLOv5	PP-PicoDet	
Scheme 1	MAE	0.37	0.39	0.39
	RMSE	0.49	0.52	0.50
	RMSPE	0.13	0.13	0.13
Scheme 2	MAE	0.37	0.39	0.39
	RMSE	0.49	0.52	0.51
	RMSPE	0.12	0.13	0.13
Scheme 3	MAE	0.35	0.39	0.38
	RMSE	0.46	0.50	0.49
	RMSPE	0.11	0.12	0.12

‘Scheme 1’ represents the BAA system bone age calculation scheme 1. ‘Scheme 2’ represents the BAA system bone age calculation scheme 2. ‘Scheme 3’ represents the BAA system bone age calculation scheme 3.MAE and RMSE units are years.

We also counted the results of various age predictions. YOLOv5 inference results after KBS, with bone age calculated for each age order, are shown in **Table 7**. PP-PicoDet inference results after KBS, with bone age calculated for each age order, are shown in **Table 8**. NanoDet inference results after KBS, with bone age calculated for each age order, are shown in **Table 9**. **Table 10** displays the statistical outcomes for both males and females. The best results were obtained using the key bone developmental grades of the YOLOv5 inference results after KBS and using Scheme 3 to get the bone age calculation model, with a MAE of 0.39 years, a RMSE of 0.50 years, and a RMSPE of 0.10 for males and a MAE of 0.32 years, a RMSE of 0.42 years, and a RMSPE of 0.11 for females.

**Table 7:** YOLOv5 inference results after KBS to calculate bone age by age group.

AGE	Scheme 3			Scheme 2			Scheme 1		
	MAE	RMSE	RMSPE	MAE	RMSE	RMSPE	MAE	RMSE	RMSPE
0-1	0.27	0.30	0.06	0.26	0.29	0.07	0.25	0.29	0.05
1-2	0.27	0.34	0.33	0.27	0.35	0.42	0.26	0.35	0.44
2-3	0.36	0.52	0.16	0.39	0.55	0.18	0.37	0.54	0.17
3-4	0.36	0.45	0.12	0.33	0.48	0.12	0.34	0.51	0.13
4-5	0.45	0.56	0.14	0.46	0.59	0.13	0.47	0.62	0.13
5-6	0.39	0.45	0.08	0.38	0.49	0.09	0.37	0.50	0.09
6-7	0.40	0.50	0.07	0.40	0.53	0.07	0.40	0.53	0.07
7-8	0.34	0.42	0.05	0.35	0.46	0.06	0.36	0.47	0.06
8-9	0.32	0.39	0.05	0.33	0.41	0.05	0.34	0.42	0.05
9-10	0.26	0.37	0.04	0.32	0.42	0.04	0.32	0.41	0.04
10-11	0.33	0.44	0.04	0.32	0.44	0.04	0.31	0.44	0.04
11-12	0.28	0.36	0.03	0.29	0.35	0.03	0.29	0.35	0.03
12-13	0.28	0.35	0.03	0.27	0.32	0.03	0.28	0.33	0.03
13-14	0.47	0.62	0.04	0.59	0.72	0.05	0.59	0.72	0.05
14-15	0.64	0.75	0.05	0.77	0.93	0.07	0.72	0.90	0.06
15-16	0.15	0.19	0.01	0.34	0.36	0.02	0.25	0.29	0.02
16-17	0.44	0.47	0.03	0.26	0.48	0.03	0.20	0.45	0.03
17-18	0.34	0.41	0.03	0.27	0.30	0.02	0.28	0.32	0.02

‘Scheme 1’ represents the BAA system bone age calculation scheme 1. ‘Scheme 2’ represents the BAA system bone age calculation scheme 2. ‘Scheme 3’ represents the BAA system bone age calculation scheme 3.MAE and RMSE units are years.

**Table 8:** Three Models inference results after KBS to calculate bone age by gender statistics.

		Scheme3		Scheme2		Scheme1	
		Male	Female	Male	Female	Male	Female
**YOLOv5**	**MAE**	0.39	0.32	0.40	0.34	0.40	0.34
	**RMSE**	0.50	0.42	0.53	0.45	0.53	0.46
	**RMSPE**	0.10	0.11	0.10	0.14	0.10	0.15
**PP-PicoDet**	**MAE**	0.40	0.37	0.41	0.38	0.40	0.38
	**RMSE**	0.53	0.48	0.55	0.50	0.54	0.50
	**RMSPE**	0.09	0.14	0.09	0.15	0.09	0.16
**NanoDet**	**MAE**	0.40	0.36	0.41	0.39	0.40	0.39
	**RMSE**	0.51	0.47	0.53	0.49	0.52	0.49
	**RMSPE**	0.10	0.13	0.11	0.14	0.11	0.14

‘Scheme 1’ represents the BAA system bone age calculation scheme 1. ‘Scheme 2’ represents the BAA system bone age calculation scheme 2. ‘Scheme 3’ represents the BAA system bone age calculation scheme 3.MAE and RMSE units are years.

**Table 9:** NanoDet inference results after KBS to calculate bone age by age group.

AGE	Scheme 3			Scheme 2			Scheme 1		
	MAE	RMSE	RMSPE	MAE	RMSE	RMSPE	MAE	RMSE	RMSPE
0-1	0.17	0.23	0.01	0.05	0.05	0.03	0.05	0.07	0.05
1-2	0.23	0.32	0.35	0.31	0.37	0.42	0.30	0.35	0.42
2-3	0.36	0.48	0.18	0.39	0.49	0.17	0.39	0.49	0.17
3-4	0.40	0.51	0.14	0.47	0.60	0.16	0.48	0.60	0.16
4-5	0.50	0.62	0.15	0.53	0.64	0.15	0.53	0.64	0.15
5-6	0.36	0.50	0.08	0.39	0.52	0.09	0.36	0.50	0.08
6-7	0.36	0.48	0.07	0.36	0.46	0.06	0.35	0.46	0.06
7-8	0.36	0.47	0.06	0.37	0.47	0.06	0.37	0.47	0.06
8-9	0.39	0.47	0.05	0.37	0.45	0.05	0.38	0.46	0.05
9-10	0.30	0.38	0.04	0.34	0.44	0.04	0.35	0.44	0.05
10-11	0.36	0.44	0.04	0.36	0.47	0.04	0.36	0.46	0.04
11-12	0.38	0.45	0.04	0.37	0.46	0.04	0.37	0.46	0.04
12-13	0.25	0.30	0.02	0.29	0.33	0.03	0.29	0.34	0.03
13-14	0.55	0.67	0.05	0.56	0.70	0.05	0.55	0.69	0.05
14-15	0.65	0.86	0.06	0.68	0.88	0.06	0.61	0.82	0.06
15-16	0.07	0.09	0.01	0.16	0.18	0.01	0.10	0.10	0.01
16-17	0.44	0.46	0.03	0.27	0.48	0.03	0.30	0.50	0.03
17-18	0.41	0.50	0.04	0.35	0.41	0.03	0.35	0.42	0.03

‘Scheme 1’ represents the BAA system bone age calculation scheme 1. ‘Scheme 2’ represents the BAA system bone age calculation scheme 2. ‘Scheme 3’ represents the BAA system bone age calculation scheme 3.MAE and RMSE units are years.

**Table 10:** Three Models inference results after KBS to calculate bone age by gender statistics.

		Scheme3		Scheme2		Scheme1	
		Male	Female	Male	Female	Male	Female
YOLOv5	MAE	0.39	0.32	0.40	0.34	0.40	0.34
	RMSE	0.50	0.42	0.53	0.45	0.53	0.46
	RMSPE	0.10	0.11	0.10	0.14	0.10	0.15
PP-PicoDet	MAE	0.40	0.37	0.41	0.38	0.40	0.38
	RMSE	0.53	0.48	0.55	0.50	0.54	0.50
	RMSPE	0.09	0.14	0.09	0.15	0.09	0.16
NanoDet	MAE	0.40	0.36	0.41	0.39	0.40	0.39
	RMSE	0.51	0.47	0.53	0.49	0.52	0.49
	RMSPE	0.10	0.13	0.11	0.14	0.11	0.14

‘Scheme 1’ represents the BAA system bone age calculation scheme 1. ‘Scheme 2’ represents the BAA system bone age calculation scheme 2. ‘Scheme 3’ represents the BAA system bone age calculation scheme 3.MAE and RMSE units are years.

BAA system speed test results on CPU 5600x showed that when the confidence threshold was set to 0.0, the rate of the YOLOv5 using KBS was 80 ms, the rate of the PP-PicoDet using KBS was 63 ms, and the rate of the NanoDet using KBS was 69 ms. When the confidence threshold was set to 0.1, the rate of the YOLOv5 using KBS was 33 ms, the rate of the PP-PicoDet using KBS was 57 ms, and the rate of the NanoDet using KBS was 61 ms. When the confidence threshold was set to 0.2, the rate of the YOLOv5 using KBS was 33 ms, the rate of the PP-PicoDet using KBS was 57 ms, and the rate of the NanoDet using KBS was 61 ms. When the confidence threshold was set to 0.3, the rate of the YOLOv5 using KBS was 33 ms, the rate of the PP-PicoDet using KBS was 57 ms, and the rate of the NanoDet using KBS was 60 ms. When the confidence threshold was set to 0.4, the rate of the YOLOv5 using KBS was 33 ms, the rate of the PP-PicoDet using KBS was 57 ms, and the rate of the NanoDet using KBS was 60 ms. When the confidence threshold was set to 0.5, the rate of the YOLOv5 using KBS was 32 ms, the rate of the PP-PicoDet using KBS was 57 ms, and the rate of the NanoDet using KBS was 59 ms. See **Table 2** for details.

All three models ran faster when the confidence threshold was raised from 0.0 to 0.1, with YOLOv5 utilizing KBS seeing the highest speed boost. And the speed did not significantly increase as the confidence threshold was raised more. When the confidence threshold rose to 0.4, all three models began to reveal missing key bones, so 0.1 was chosen as the ideal confidence threshold for KBS. The YOLOv5 model with KBS was the fastest when comparing the three models with confidence thresholds greater than 0.1. This is because the outputs of the YOLOv5 result are all possible positions, the confidence level corresponding to the position, and the category vector corresponding to the position, which means that using confidence thresholds can first screen out the results with lower box confidence, greatly enhance speed. In contrast, the outputs of PP-PicoDet and NanoDet are all possible positions, and the category vector corresponds to the position, which means that using confidence thresholds should first process the classification vectors to obtain the result categories and confidence levels. The second part of the system chose to use Lightgbm to calculate bone age with an average elapsed time of 2 ms. Finally, the first part of the system was selected as YOLOv5, the confidence threshold of KBS was selected as 0.1 and pre-processing was performed with the help of the Albumentations (36). The average processing time in the environment of GPU RTX3060 is 26 ms.

## 4 Discussion

In this paper, we propose a BAA system based on the RUS-CHN method, which is based on real-time target detection and obtaining the developmental grades and locations of the key bone of RUS-CHN in a single step with the assistance of KBS, then using Lightgbm to obtain the bone age, capable of completing real-time outputs. While most of the current work, is based on the morphological features of wrist bone or RUS bone to extract reference or region (finger bone, etc.) as local information input, extracting ROI is still complex and time-consuming, and selective extraction of regions is not objective enough leading to some key information loss (37).

The system is developed from 3628 training sets, 450 validation sets, and 450 test sets of clinical hand radiographs, incorporating data with an age distribution covering infancy to late adolescence, so that our system has high accuracy and stability in BAA for young children and older adolescents. To use it without hand shape segmentation, we also forgo hand shape extraction and instead CLAHE. The achieved real-time outputs with an inference time of 26ms + 2ms on the GPU, which is significantly faster than the average time of 525.6s ± 55.5s (15) required by endocrinologists or radiologists to assess bone age using the RUS-CHN method. The system has a MAE of 0.35 years and a RMSE of 0.46 years, a RMSPE of 0.11. The system outputs not only the predicted bone age but also the location and developmental grade of all critical bones to support the results as shown in **Figure 5B**, where the IOU of all key bone locations is not less than 0.9.

Also, our system has several restrictions. All photographs were gathered from our hospital, and more images will be uploaded in the future to decrease bias from other medical centers. Additionally, attempt more clinical *a priori* procedures, such as the TW and CHN methods. Second, specific disorders, such as renal osteodystrophy and chondrodysplasia, cannot be detected by our approach in youngsters (38).

## 5 Conclusions

We have developed an automated end-to-end BAA system that is based on real-time target detection, obtaining key bone developmental grade and location in a single pass with the aid of KBS, and using Lightgbm to obtain bone age, capable of outputting results in real-time with good accuracy and stability, and able to be used without hand-shaped segmentation. The BAA system automatically implements the entire process of the RUS-CHN method and outputs information on the location and developmental grade of the 13 key bones of the RUS-CHN method along with the bone age to assist the physician in making judgments, making full use of clinical *a priori* knowledge. This system can free clinicians from the tedious clinical observation process and ultimately improve children’s diagnosis and treatment of endocrine diseases.

## Data availability statement

The original contributions presented in the study are included in the article/supplementary material. Further inquiries can be directed to the corresponding authors.

## Ethics statement

The studies involving human participants were reviewed and approved by The ethical review board of Medical Ethics Committee of Children’s Hospital of Chongqing Medical University in accordance with the principles of Declaration of Helsinki (NO: 2022-IRB-151). The data are anonymous, and the requirement for informed consent was therefore waived. Written informed consent from the participants’ legal guardian/next of kin was not required to participate in this study in accordance with the national legislation and the institutional requirements.

## Author contributions

CY drafting the article, building the deep learning model, experimental analysis, and experimental design is the main contributor to writing the manuscript. WD helps with data cleaning and result collection. BQ organizes training staff to label patient images. XH and WZ reviewed the manuscript. All authors read and approved the final manuscript.
